# Exertional heat stroke in a young military trainee: is it preventable?

**DOI:** 10.1186/s40779-016-0078-1

**Published:** 2016-03-31

**Authors:** Buddhika T. B. Wijerathne, Senaka D. Pilapitiya, Vadivel Vijitharan, Mohammed M. F. Farah, Yashodhara V. M. Wimalasooriya, Sisira H Siribaddana

**Affiliations:** Department of Forensic Medicine, Faculty of Medicine and Allied Sciences, Rajarata University of Sri Lanka, Saliyapura, Sri Lanka; Department of Medicine, Faculty of Medicine and Allied Sciences, Rajarata University of Sri Lanka, Saliyapura, Sri Lanka

**Keywords:** Heat stroke, Military trainee, Asymptomatic infection

## Abstract

**Background:**

Heat stroke is a life-threatening condition with exertional heat stroke occurring frequently among soldiers and athletes. Because of its common occurrence, many military trainees practice preventive measures prior to any activity requiring severe exertion. Although it is said to be common in practice, different presentations of heat stroke are scarcely described in literature.

**Case Presentation:**

We describe a case of an exertional heat stroke in a 23-year-old male Sinhalese soldier who developed early changes of renal failure, liver failure and rhabdomyolysis. The patient initially presented with convulsions, delirium and loss of consciousness to an outside health care facility before being transferred to our institution.

**Conclusion:**

It is clear that heat stroke does occur in military trainees while preventive strategies are being practiced. It is important for those who provide healthcare to soldiers to provide proper advice on how to identify impending heat stroke prior to any exercises resulting in severe physical exertion. Further, treating physicians should educate all military trainees about preventive strategies.

## Background

Heat stroke is described as an elevated core body temperature of more than 40 °C with central nervous system (CNS) dysfunction, causing subsequent delirium, convulsions or coma [1].

There are two types of heat stroke, classical and exertional [2, 3]. Classical heat stroke is generally caused by high environmental temperature, develops slowly over a few days, and can present with delirium, convulsions or coma [1, 2]. This condition mainly occurs in elderly people debilitated with chronic illness [4]. Exertional heat stroke primarily affects young and active people such as marathon runners, athletes, or military personnel [5–7]. Intrinsic heat production is the main underlying cause [8].

We report a case of heat stroke in a young military trainee who was aware of the potential for heat stroke during exertion, and had taken precautions to avoid it.

## Case Presentation

On the evening of May 23^rd^ 2015, a 23-year-old military trainee was transferred from the District General Hospital Mannar (DGHM) (situated in the dry zone of Sri Lanka) to the Teaching Hospital Anuradhapura (THA) for further management of coma and convulsions. The patient was participating in a 9 mile ruck marching marathon on that same morning. After about four hours, the patient fainted and had a seizure near the finishing line. His companions cooled his body with water and brought him to the DGHM at approximately 9 a.m. that morning.

After admission to DGHM, he regained partial consciousness in a few minutes and was hyperactive, irritable, confused and amnesic. His Glasgow Coma Scale (GCS) was 9, and his temperature was 39 °C, pulse was 119 beats per minute, and blood pressure was 115/57 mmHg. His arterial blood gas showed a partially-compensated metabolic acidosis [pH of 7.34 (7.35-7.45), P_a_CO_2_ of 27 (35–45) mmHg, HCO_3_^−^ of 14.6 (32–26) mEq/L], a blood glucose of 66 mg/dl, a serum creatinine of 2 mg/dl, and normal electrolytes, including calcium. He had a high alanine aminotransferase (ALT) (114 U/L), Aspartate aminotransferase (AST) (104 U/L) and white blood cell (WBC) count with granulocyte predominance (granulocytes were 74.8 %, and lymphocytes were 11.5 %). He was given intravenous diazepam 5 mg, and ceftriaxone 2 g/day was initiated. Rapid cooling was initiated with cool water and the use of hand fans. He regained full consciousness two hours after admission to the THA (12 h after the incident).

The previous day, the patient was asymptomatic. He had not completed any special preparation before the marathon and only drank water to avoid possible heat stroke. He was carrying a backpack weighing nearly 10 kg, which also included a weapon. It was a sunny day (May 23^rd^, 2015) with an average temperature during the day of 30 °C, no rain, 17 km/h wind speed and 76 % humidity (http://www.wunderground.com). The patient fainted while he was sprinting to the finish line. He reported that his face felt warm, and he had less sweating compared to his previous runs during his training period. He also felt pain in his thighs while running. He drank water before and during the run. He fully regained his awareness in THA nearly 12 h after the incident. He only failed to recall anything that occurred just after the fall.

His liver function tests reached the highest values on the fourth day of his hospital stay (ALT of 408 U/L and AST of 601 U/L). On the eighth day after admission, ALT and AST decreased to values of 132 U/L and 86 U/L, respectively, and became normal by the time of discharge on the tenth day. His clotting profile was normal. Acute renal failure was evident from the day of admission (Serum creatinine of 2 mg/dl, e-GFR of 46 ml/min/1.73 m^2^ (CKD-EPI)] to the third day. Timely fluid management halted further decline of renal function. His neurological examination and computed tomography scan were normal.

His blood count on the second day of his hospital stay showed a white blood cell count of 10.2 × 10^9^/L with neutrophilia (neutrophils were 78.6 % and lymphocytes were 11.6 %); this normalized by the ninth day of admission. On the third day, he had mild bilateral thigh pain and tenderness. His creatinine phosphokinase was 17700 U/L. The results of urine myoglobin testing were unavailable due to laboratory error. However, his urine color was normal throughout his hospital stay.

**Fig. 1:**
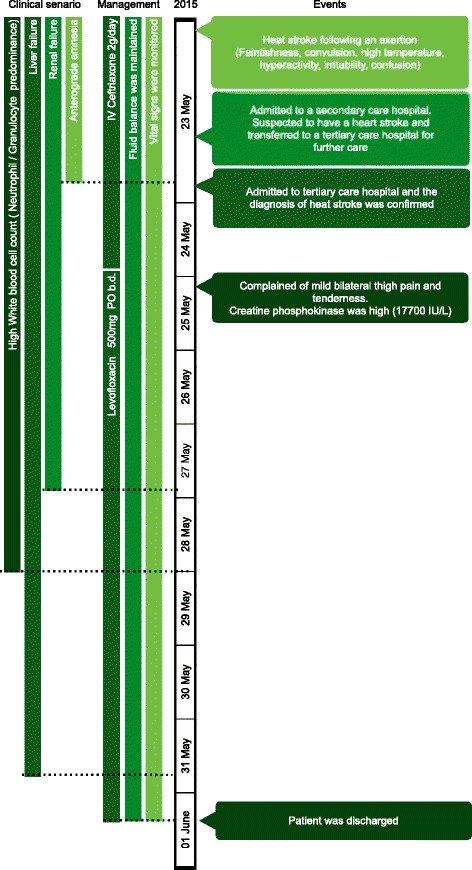
Timeline of clinical presentations, managements and outcomes of the patient from the beginning of the heatstroke to full recovery

He was given intravenous fluid (0.9 % saline) for the first three days. Ceftriaxone was changed to oral levofloxacin 500 mg bid after the second day. The patient was discharged on the tenth day. The timeline diagram shows the sequence of events (clinical presentations, complications, management) from the time of heat stroke until discharged from the hospital (Fig. 1).

## Discussion

The patient developed heat stroke during strenuous physical activity in a hot and humid environment. His recorded temperature was 39 °C after cooling. Though treatment had been initiated for heat stroke, CNS changes were evident. His retrograde amnesia may have been related to diazepam given for his seizure. The heat stroke was associated with acute kidney injury, liver failure, and probable rhabdomyolysis.

His high granulocyte and neutrophil counts in the initial bloodwork suggested asymptomatic non-febrile bacterial infection prior to onset of the heat stroke. Underlying infection might have decreased his immunity while increasing his metabolic rate, subsequently augmenting the heat stroke. Several reports note that an acute inflammatory response could increase susceptibility to exertional heat stroke [3, 5]. Several theories describe possible mechanisms of action that demonstrate how pre-existing infections can precipitate heat stroke. Cytokines (IL-6, IL-1 beta, and INF-gamma) seems to be the major culprit [9]. Sonna and others postulated that pro-inflammatory cytokines generated in previous infections deactivate the ability of cells to protect themselves from extremely high temperatures [10]. Roberts reported an occurrence of heat stroke in a well-trained male runner in cool weather with a viral infection the week prior [11]. Sonkar and others described a severe heat stroke in a patient who also had a high neutrophil count [12]. A full blood count carried out at the commencement of the training (2 weeks before the incidence) was normal. However, we cannot confirm a bacterial infection based on a high white cell count with predominant granulocytes [13]. Nonetheless, clinicians should suspect the presence of underlying infection when adult patients develop heat stroke without any other precipitating causes. At the DGHM, IV antibiotics were begun on the suspicion of cerebral infection. He did not show any clinical signs of infection in the next few days. Therefore, IV antibiotics were continued until the end of the second day, and then they were changed to oral levofloxacin. The advantages of early discontinuation of IV antibiotics have been well described [14, 15]. The outcome of our patient was not affected by this intervention.

Similar presentations of patients in the same age group and similar environment were described in previous reports. However, the complications they developed were more severe. Wakino and others reported a heat stroke in a 23-year-old male construction worker (comatose with low GCS) who was working in a hot (28.1 °C) and humid (53 %) environment and developed multi-organ dysfunction [16]. Gómez Ramos and others described a fatal heat stroke in a 20-year-old male with schizophrenia, hypertension and obesity working in 30 °C heat who also developed multi-organ dysfunction [17]. However, our patient’s comorbidities were less significant, even though he did exert himself in a similar environment (temperature 30 °C and humidity 76 %). This may be due to early medical care.

Our patient was well aware of the potential of heat stroke during military training and the need for adequate hydration before strenuous physical activity. However, he was not aware of the clinical features. He experienced hot flashes with less sweating just a few minutes prior to loss of consciousness. Appropriate health education should include recognizing impending heat stroke, its clinical course, the provision of first aid to high-risk groups, and noting that military trainees work in hot and humid environments. Finally, the treating physician should consider the possibility of underlying infection in cases of exertional heat stroke presenting without known precipitating factors.

## Conclusions

It is evident that, even though preventive strategies are employed, heat stroke does occur in military exercise. In our patient, possible asymptomatic bacterial infection could have been the factor that enhanced the development of heatstroke, but this is currently unconfirmed. It is important for health care providers of soldiers and athletes to monitor for infection and provide proper advice on how to identify impending heat stroke, prior to any events resulting in severe physical exertion. Furthermore, the treating physician should educate all military trainees about preventive strategies.

### Consent

Written informed consent was obtained from the patient for publication of this case report. A copy of the written consent is available for review by the Editor-in-Chief of this journal.

## Abbreviations

ALT: Alanine transaminase
AST: Aspartate aminotransferase
CNS: Central nervous system
DGHM: District general hospital mannar
GCS: Glasgow coma scale
IV: Intravenous
THA: Teaching hospital Anuradhapura
WBC: White blood cells
